# Long term follow up of biomarkers of podocyte damage and renal function in patients with and without preeclampsia

**DOI:** 10.1590/2175-8239-JBN-3941

**Published:** 2018-05-17

**Authors:** Ylbe Palacios de Franco, Karina Velazquez, Natalia Segovia, Gladys Sandoval, Estefania Gauto, Ylbe V. Franco Palacios, Carlos R Franco Palacios

**Affiliations:** 1 Universidad Católica Nuestra Señora de la Asunción, Facultad de Ciencias de la Salud, Asunción, Paraguay.; 2 Instituto de Prevision Social, Departamento de Medicina de Laboratorio, Asuncion, Paraguay.; 3 Instituto de Prevision Social, Departamento de Inmunología Clínica, Asuncion, Paraguay.; 4 Instituto de Prevision Social, Departamento de Educación Médica, Asuncion, Paraguay.; 5 South Jersey General Hospital, Department of Obstetrics and Gynecology, Vineland. NJ, USA.; 6 Rice Memorial Hospital, Department of Nephrology, Affiliated Community Medical Centers, Willmar, MN, USA.

**Keywords:** Hypertension, Kidney Diseases, Podocytes, Hipertensão, Doenças Renais, Podócitos

## Abstract

**Introduction:**

preeclampsia can be associated with future renal disease.

**Objectives:**

To measure changes in renal function overtime in patients with preeclampsia.

**Methods:**

urine and serum samples from eleven patients with preeclampsia and eight patients with a normal pregnancy were obtained during pregnancy, postpartum, and 3 years after delivery. Urine podocalyxin, protein, and serum creatinine were measured.

**Results:**

after 3 years, there were no significant differences in urinary podocalyxin in patients with or without preeclampsia: 4.34 ng/mg [2.69, 8.99] *vs*. 7.66 ng/mg [2.35, 13], *p* = 0.77. The same applied to urinary protein excretion: 81.5 mg/g [60.6, 105.5] *vs*. 43.2 mg/g [20.9, 139.3] *p* = 0.23. Serum creatinine was 0.86 mg/dL [0.7, 0.9] *vs*. 0.8 mg/dL [0.68, 1] *p* = 0.74 in those with and without preeclampsia. In normal patients, urinary podocalyxin decreased from 54.4 ng/mg [34.2, 76.9] during pregnancy to 7.66 ng/mg [2.35, 13] three years after pregnancy, *p* = 0.01. Proteinuria decreased from 123.5 mg/g [65.9, 194.8] to 43.2 mg/g [20.9, 139.3], *p* = 0.12. In preeclampsia patients, urinary podocalyxin decreased from 97.5 ng/mg [64.9, 318.4] during pregnancy to 37.1 ng/mg within one week post-partum [21.3, 100.4] *p* = 0.05 and 4.34 ng/mg [2.69, 8.99] three years after, *p* = 0.003. Proteinuria was 757.2 mg/g [268.4, 5031.7] during pregnancy *vs*. 757.2 mg/g [288.2, 2917] postpartum, *p* = 0.09 *vs*. 81.5 mg/g [60.6, 105.5] three years later, *p* = 0.01. Two patients still had proteinuria after 3 years.

**Conclusions:**

in preeclampsia patients, postpartum urinary podocalyxin decreased before proteinuria. After three years, serum creatinine, urinary podocalyxin, and protein tended to normalize, although some patients still had proteinuria.

## RESUMO

**Introdução:**

a pré-eclâmpsia pode estar associada à doença renal no futuro. Objetivos: medir mudanças na função renal ao longo do tempo em pacientes com pré-eclâmpsia.

**Métodos:**

amostras de urina e soro de onze pacientes com pré-eclâmpsia e oito pacientes com gravidez normal foram obtidas durante a gravidez, pós-parto e 3 anos após o parto. Medimos podocalixina na urina, proteína e creatinina sérica.

**Resultados:**

após 3 anos, não houve diferenças significativas na podocalixina urinária em pacientes com ou sem pré-eclâmpsia: 4,34 ng/mg [2,69, 8,99] versus 7,66 ng/mg [2,35, 13], *p* = 0,77. O mesmo se aplicou à excreção urinária de proteínas: 81,5 mg/g [60,6, 105,5] *vs*. 43,2 mg/g [20,9, 139,3] *p* = 0,23. A creatinina sérica foi de 0,86 mg/dL [0,7, 0,9] *vs*. 0,8 mg/dL [0,68, 1] *p* = 0,74 naqueles com e sem pré-eclâmpsia. Em pacientes normais, a podocalixina urinária diminuiu de 54,4 ng/mg [34,2, 76,9] durante a gestação para 7,66 ng/mg [2,35, 13] três anos após a gravidez, *p* = 0,01. A proteinúria diminuiu de 123,5 mg/g [65,9, 194,8] para 43,2 mg/g [20,9, 139,3], *p* = 0,12. Em pacientes com pré-eclâmpsia, a podocalixina urinária diminuiu de 97,5 ng/mg [64,9, 318,4] durante a gravidez para 37,1 ng/mg em uma semana de pós-parto [21,3, 100,4] *p* = 0,05 e 4,34 ng/mg [2,69, 8,99] três anos depois, *p* = 0,003. A proteinúria foi de 757,2 mg/g [268.4, 5031.7] durante a gravidez *vs*. 757,2 mg/g [288.2, 2917] pós-parto, *p* = 0.09 *vs*. 81.5 mg/g [60.6, 105.5] três anos depois, p = 0.01. Dois pacientes ainda apresentavam proteinúria após 3 anos.

**Conclusões:**

em pacientes com pré-eclâmpsia, a podocalixina urinária pós-parto diminuiu antes da proteinúria. Após três anos, a creatinina sérica, a podocalixina urinária e a proteína tenderam a se normalizar, embora alguns pacientes ainda tivessem proteinúria.

## INTRODUCTION

Preeclampsia affects 3-10% of pregnancies and its prevalence has been increasing. This condition is a significant risk factor for maternal death, perinatal death, preterm birth, and low birthweight.^1^^,^ 2

Older maternal age, low socioeconomic status, obesity, anemia, nulliparity, lack of prenatal care, hypertension, diabetes, and chronic kidney disease among others are risk factors for the development of the disease.^2^

Over the years, there has been an active search for biomarkers of preeclampsia that could improve its diagnosis. Currently, no recommended screening tests are available besides taking a thorough medical history to identify potential risk factors for the disease.^3^

Podocytes are normally absent or seen in small numbers in the urine of healthy individuals or those with inactive kidney disease. In a prior study, we found significantly higher levels of urinary podocalyxin (a marker of urinary podocyte loss -podocyturia) in preeclampsia/eclampsia patients, with a tendency to normalize after delivery.^4^

It has been postulated that preeclampsia can be associated with future cardiovascular and renal disease. For instance, patients with hypertension during pregnancy a greater subsequent risk of microalbuminuria than those with normotensive pregnancies.^5^

After preeclampsia, it can take up to 2 years for hypertension and proteinuria to resolve. Because the presence of persistent post-partum urinary podocyte excretion (podocyturia) and proteinuria in patients who suffered preeclampsia might indicate ongoing subclinical renal damage, we measured renal function, proteinuria, and urinary podocalyxin in these patients 3 years postpartum.

To the best of our knowledge, this is the first study measuring urine biomarkers in these patients for a longer period of time. We hypothesized that patients with a history of preeclampsia have ongoing subclinical renal damage that could explain their increased risk of chronic kidney disease later in life, and this could be demonstrated by persistent podocyte loss in the urine.

## MATERIAL AND METHODS

With Institutional Review Board approval, this prospective observational study was performed between March 2013 and May 2016.

*Inclusion criteria*: after obtaining informed consent, patients who were pregnant in 2013 and received care at the Obstetrics service at the IPS (Instituto de Prevision Social) Hospital in Asuncion, Paraguay were enrolled in this study. They were followed up for 3 years after pregnancy.

Urine and blood tests were obtained at baseline (during pregnancy), within one week post-partum (in those with preeclampsia), and three years later.

Mild preeclampsia was defined as a new development of hypertension (SBP > 140/90 mm Hg) on 2 occasions at least 6 hours apart in a woman without evidence of chronic hypertension and who was normotensive before 20 weeks of gestation, along with proteinuria ≥ 300 mg.

*Severe preeclampsia* was defined as preeclampsia complicated by either a SBP ≥ 160 mm or a DBP ≥ 110 mm Hg on 2 occasions at least 6 hours apart, and/or pulmonary edema, and/or oliguria (< 400 mL of urine output in 24 hours), and/or persistent headaches and neurological symptoms, and/or epigastric pain, and/or impaired liver function, and/or thrombocytopenia, and/or oligohydramnios, decreased fetal growth or placental abruption, and/or HELLP syndrome (hemolysis, elevated liver enzyme, low platelets).

Eclampsia was defined as seizures that cannot be attributable to other causes in a woman with preeclampsia.

Normal pregnancy control*:* patients without a diagnosis of preeclampsia or eclampsia. Patients without the conditions listed in the exclusion criteria.

*Exclusion criteria:* patients without available urine or blood tests at the time of the study, patients with prior history of chronic kidney disease, glomerulonephritis, hematuria, autoimmune disorders, cancer or diabetes mellitus, and patients pregnant at the time of follow up.

*Variables collected:* age, serum creatinine, urine protein, urine podocalyxin, and urine creatinine.

*Estimation of podocyturia*: random urine (10 mL) was collected in plastic tubes without preservative. If necessary, they were clarified by centrifugation (at 3,000 rpm for 5 min). The urine was kept at 4ºC for up to 1 week. Prior to the assay, the samples were allowed to thaw at room temperature (24ºC). All the assays were completed using duplicate wells for each dilution of the standard and of the sample.

A commercially available podocalyxin ELISA test (Exocell Inc.) was used. This assay is designed to measure podocalyxin in urine or tissue samples of rodent or human origin. The assay range is 0.156-10.0 ng/mL. The intra- and inter-assay precision for samples within the assay range have a CV of < 7%. Each sample was measured in duplicate. The values are expressed as ng/mL.

*Estimation of renal function:* renal function was expressed as serum creatinine (mg/dL).

*Estimation of proteinuria*: random urine total protein-to-creatinine ratio (UPC, expressed as mg/g) was obtained. Total protein was measured by the Pyrogallol red dye method. Urine creatinine was measured by the Jaffe reaction on the same aliquot of urine. To further adjust for urine creatinine, the ratio of urinary podocalyxin to creatinine (ng/mg) was used.

Statistical analyses: data are presented as mean and standard deviation if normally distributed and median [25 and 75% percentiles] if not.

Differences in means were compared by the Student's t test. For highly skewed data, the Mann-Whitney U test was used. For paired data, the Wilcoxon signed rank test was used.

Differences in proportions were assessed by the Fisher's exact test.

P values lower ≤ 0.05 were considered statistically significant. All the analyses were performed using SOFA Statistics version 1.4.0 (Paton-Simpson & Associates Ltd, Auckland, New Zealand) and JMP statistical software version 11.2.0 (SAS Campus Drive, Cary, NC).

## RESULTS

The baseline characteristics are depicted in Table 1. Patients with preeclampsia tended to have higher levels of urinary podocalyxin and protein during pregnancy.

**Table 1:** Baseline characteristics during pregnancy. Patients with preeclampsia tended to have more proteinuria and podocyturia (urinary podocalyxin)

	Normal pregnancyN = 8	PreeclampsiaN = 11	p value
Age (years), mean ± SD	32.2 ± 5.11	29.8 ± 5.86	0.31
Gestational age (weeks), median [IQR]	37 [36.2, 38.7]	34 [30, 36]	0.06
Serum creatinine (mg/dL), median [IQR]	0.6 [0.59, 0.86]	0.74 [0.5, 1.08]	0.77
Urine protein/creatinine (mg/g), median [IQR]	123.5 [65.9, 194.8]	757.2 [268.4, 5031.7]	0.001
Urine podocalyxin/creatinine (ng/mg), median [IQR]	54.4 [34.2, 76.9]	97.5 [64.9, 318.4]	0.09

IQR = interquartile range. SD = standard deviation.

**Figure 1:**
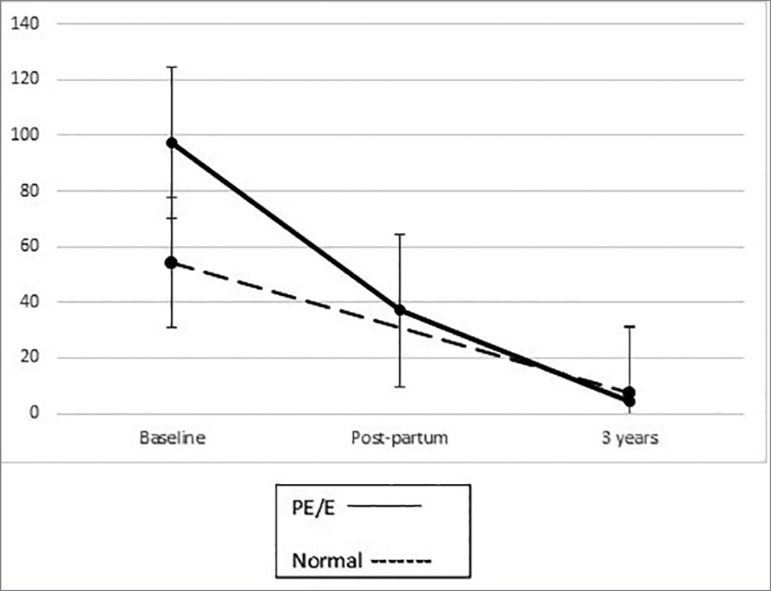
Urinary podocalyxin (ng/mg) overtime. In both groups, urinary podocalyxin decreased after pregnancy. After 3 years, there was no difference in podocyturia between groups (bars are standard error).

**Figure 2:**
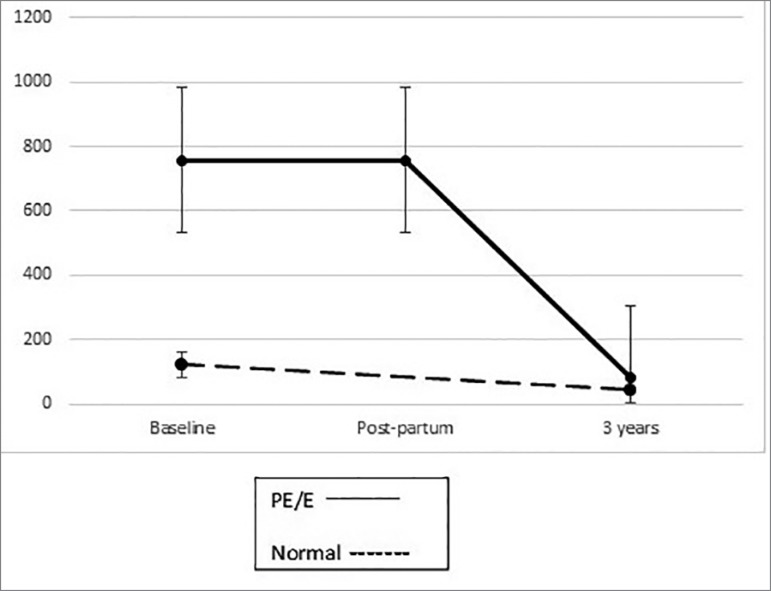
Proteinuria (mg/g) overtime. In both groups, proteinuria decreased after pregnancy. After 3 years, there was no difference in proteinuria between groups (bars are standard error).

Urinary levels of podocalyxin and protein decreased overtime, in both groups. (Figure 1 and 2).

After 3 years, there was no significant difference in urinary podocalyxin in patients with or without preeclampsia: 4.34 ng/mg [2.69, 8.99] *vs*. 7.66 ng/mg [2.35, 13], *p* = 0.77. The same applies to urinary protein excretion: 81.5 mg/g [60.6, 105.5] *vs*. 43.2 mg/g [20.9, 139.3] *p* = 0.23.

**Figure 3:**
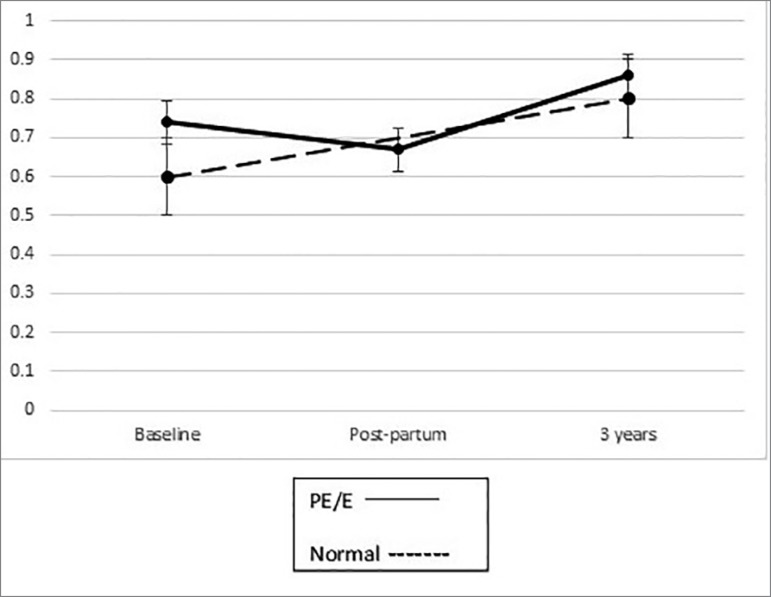
Serum creatinine (mg/dL) overtime. After 3 years, no difference in renal function was noted between groups (bars are standard error).

At 3 years, the serum creatinine was 0.86 mg/dL [0.7, 0.9] *vs*. 0.8 mg/dL [0.68, 1] *p* = 0.74 in those with and without preeclampsia respectively (Figure 3).

In normal patients, urinary podocalyxin decreased from 54.4 ng/mg [34.2, 76.9] during pregnancy to 7.66 ng/mg [2.35, 13] three years after pregnancy, *p* = 0.01. Proteinuria fell from 123.5 mg/g [65.9, 194.8] to 43.2 mg/g [20.9, 139.3], *p* = 0.12.

In patients with preeclampsia, urinary podocalyxin decreased from 97.5 ng/mg [64.9, 318.4] during pregnancy to 37.1 ng/mg within one week post-partum [21.3, 100.4] *p* = 0.05 and 4.34 ng/mg [2.69, 8.99] three years after pregnancy, *p* = 0.003. Proteinuria was 757.2 mg/g [268.4, 5031.7] during pregnancy *vs*. 757.2 mg/g [288.2, 2917] postpartum, *p* = 0.09 *vs*. 81.5 mg/g [60.6, 105.5] three years later, *p* = 0.01. Urinary levels of podocalyxin decreased faster than proteinuria.

Two patients with a history of preeclampsia still had proteinuria (urine protein to creatinine ratio > 300 mg/g) at 3 years, *vs*. none in the normal pregnancy group. In these two proteinuric patients, postpartum urinary podocalyxin was 37.1 ng/mg and 182.9 ng/mg respectively; after 3 years it was 1.81 and 2.67 ng/mg. Postpartum proteinuria was 28.3 mg/g and 94.6 mg/g.

## DISCUSSION

Several studies suggest that women with hypertensive disorders during pregnancy have a greater risk of chronic kidney disease, hypertension, venous thromboembolism, and type 2 diabetes mellitus even after controlling for common risk factors. Furthermore, women with preeclampsia or eclampsia might have a higher risk of end-stage renal disease compared to women who had gestational hypertension only.^6^^,^ 7

The cardiovascular and renal risks in preeclampsia patients might be due to the presence of anti-angiogenic factors, like the soluble vascular endothelial growth factor receptor 1 (sVEGFR-1) also known as soluble fms-like tyrosine kinase 1 (sFlt1). The effects of proangiogenic factors such as VEGF (vascular endothelial factor) and PIGF (placental growth factor) are antagonized by sFlt-1. Increased levels of sFlt-1 and reduced levels of PlGF might predict the subsequent development of preeclampsia.^8^^,^ 9

In addition to the endothelial dysfunction present in preeclampsia, podocyte damage occurs. Prior studies have shown that podocyturia could be used as a marker of preeclampsia, and might be more specific and sensitive than anti-angiogenic markers in diagnosing the disease.^10^^,^ 11 Different markers of urinary podocyte excretion have been used, among them nephrin and podocalyxin. These proteins are elevated in preeclampsia and barely detectable in normal women and women with chronic hypertension.^12^ On the other hand, podocyturia has been detected in the urinary sediments of patients with various forms of glomerulonephritis, particularly those with acute onset.^13^

Even after delivery, some alterations persist in patients affected with preeclampsia. Women might have podocyturia 5-8 weeks post-partum, and 39% still have hypertension after 3 months, which decreases to 18% after 2 years. Three months postpartum, 14% have proteinuria, which decreases to 2% after 2 years.^14^^,^ 15 In theory, such persisting markers of ongoing renal injury (i.e urinary podocalyxin, proteinuria) would signal patients at higher risk of progressive kidney disease.

Similarly to other studies, in this cohort we found that both urinary podocalyxin and protein levels are higher during pregnancy in those with preeclampsia. They tend to normalize after delivery, although we noted that two patients with preeclampsia still had proteinuria after 3 years.

In patients with preeclampsia, podocyturia at 3 years was lower than during pregnancy and postpartum and was similar to patients without preeclampsia. Serum creatinine was similar in the two groups as well upon follow up. Our findings are in line with prior studies that suggest that podocyturia tends to be limited to ongoing glomerular damage, and it disappears after the underlying condition is corrected. In contrast to this, proteinuria can be a marker of ongoing glomerular disease or chronic glomerular injury and it might take longer to normalize.^16^

It is important to note that the patients in our study were young and had no comorbid conditions upon enrollment. We were unable to measure anti-angiogenic factors.

This study had a long follow up and found no differences in renal function in most patients after 3 years. It seems that the renal damage caused by preeclampsia is most noticeable during the course of the disease and resolves in the majority of the patients, although proteinuria can be present in few cases even after 3 years.

This was a small cohort and further studies with larger samples are required for final conclusions. Future studies should include serum and urinary markers of preeclampsia measured at different time points.

## CONCLUSION

In preeclampsia, postpartum urinary podocalyxin decreased before proteinuria. After three years, serum creatinine, urinary podocalyxin, and urinary protein tend to normalize, although some patients with preeclampsia can still present proteinuria.

## DECLARATIONS

**Ethical considerations:** all procedures performed in studies involving human participants were in accordance with the ethical standards of the institutional and/or national research committee and with the 1964 Helsinki declaration and its later amendments or comparable ethical standards.
